# Overweight/obesity and socio-demographic disparities in children’s motor and cognitive function

**DOI:** 10.3389/fpsyg.2023.1134647

**Published:** 2023-05-23

**Authors:** Samantha Moss, Xiaoxia Zhang, Priscila Tamplain, Xiangli Gu

**Affiliations:** ^1^Kinesiology Department, State University of New York at Cortland, Cortland, NY, United States; ^2^Kinesiology Department, Centenary College of Louisiana, Shreveport, LA, United States; ^3^Kinesiology Department, University of Texas at Arlington, Arlington, TX, United States

**Keywords:** obesity, cognitive function, fundamental motor skills, socio-economic status, preschoolers, reaction time, movement time

## Abstract

Fundamental motor skills (FMS) and cognitive function are important indicators of development in early childhood. Using a cross-sectional design, the purpose of this study was to investigate obesity (healthy weight vs. overweight/obese) and socio-demographic (i.e., gender, SES) disparities in FMS (locomotor and ball skills) and cognitive function (reaction time [RT] and movement time [MT]), in preschoolers. There were 74 preschoolers (girl = 38; M_age_ = 4.02 ± 0.73) recruited from two childcare centers and were categorized into healthy weight (*n* = 58, BMI percentile < 85%) and overweight/obese (*n* = 16, BMI percentile ≥ 85%) categories. Children’s FMS were assessed using the TGMD-3; cognitive function was assessed by the iPad-based CANTAB™ software using the Reaction Time Task (RTI), including reaction time (RT; Simple RT [SRT], choice RT [CRT]) and movement time (MT; simple MT [SMT], choice MT [CMT]). Children presented less FMS proficiency compared to recent data. Both weight groups performed comparably in FMS (*p*s > 0.05; ball skill Cohen’s *d* = 0.40; locomotor Cohen’s *d* = 0.02). Children in the overweight/obese group performed significantly worse on all cognitive tests compared to healthy weight peers (*p*s < 0.05; Cohen’s *d* range from −0.93 to −1.43). No significant gender or SES disparities were observed. Maintaining healthy weight status is critical for cognitive development among preschoolers, which may influence their developmental trajectory and school readiness.

## 1. Introduction

According to the recent report from the Center for Disease Control and Prevention, over 45 million children aged 2–5 years old are categorized as overweight/obese in the U.S. which has increased by almost 4 million children over the last 6 years (CDC Division of Nutrition, 2021). Children categorized as overweight/obese pose themselves at a higher risk for developmental delays and poorer fundamental school readiness skills compared to their healthy weight counterparts (Kelsey et al., 2014; Pearce et al., 2016). A recent meta-analysis included 52 studies and noted that children and adolescents (5–18 years old) that were obese were 1.4–26.1 times more likely to have metabolic comorbidities and/or psychological issues than healthy weight peers (Sharma et al., 2019). This evidence widens the need for more research in the early childhood stage (3–5 years old) in order to establish a clearer consensus on the effects of weight status on other developmental and health-related outcomes. Research has consistently reported how obesity contributes to health and educational disparities and its association with poorer academic performance in childhood, especially in children with a disadvantaged and low socioeconomic status (Winter and Sass, 2011). The evidence suggests that obesity may have detrimental effects on development trajectory and related indicators such as fundamental motor skills (FMS) and cognitive functioning, especially during early childhood (Khalaj and Amri, 2013; Chang and Gu, 2018; Eichen et al., 2018).

Early childhood is a crucial time for assessing and augmenting specific developmental skills and potential social determinants, especially given how health disparities have been further enlarged during the COVID-19 pandemic (Krombholz, 2013; Martin et al., 2016; López-Bueno et al., 2020). FMS are goal-derived basic gross motor skills that form specific skilled movements and functioning for daily activities (Kipling Webster and Ulrich, 2017). Proficiency in FMS can be used as an indicator to gauge children’s cognitive, social, and emotional development (McClelland and Cameron, 2019; Viegas et al., 2021). FMS including locomotor skills (e.g., running, galloping, jumping, hopping, and sliding) and ball skills (e.g., catching, throwing, kicking, and dribbling) are introduced and learned in early childhood years (Lloyd et al., 2014; Kipling Webster and Ulrich, 2017). Mastery of these movements through learning, practice, and reinforcement is essential for a child’s independence and positive developmental trajectory in physical, motor, and cognitive health as they mature (Logan et al., 2012). Children that demonstrate motor delays are also likely to incur cognitive and academic struggles (Cameron et al., 2012). A previous literature review synthesized research in preschoolers, children, adolescents, and adults infered underperformance in academics are ingrained from attention deficit hyperactivity disorder (ADHD) symptoms and underlying cognitive deficits (Daley and Birchwood, 2010). Daley and Birchwood also suggested that enhancing children’s cognitive function and providing tools for motor enhancement (fidget spinner, stress ball) could be helpful strategies to indirectly improve academic performance. Ultimately, it’s important to assess children’s motor competency and cognitive function in early childhood so potential developmental delays are monitored and appropriate actions are taken early on.

Obesity/overweight has also been investigated as a potential indicator for developmental delays in children. From a socio-ecological perspective, Hulteen et al. (2018) proposed a conceptual model supporting the notion that skill development may be impacted (hindered or enhanced) by weight status along with other physical and psychological attributes from infant to adult. From this model, it would be of importance to investigate influences of weight status on FMS and cognitive function in a young childhood sample. Evidence of cognitive differences based on weight status has been noted in the literature, but mostly in elementary-aged children (6–11 years old), so weight status and cognition have not been adequately investigated in a young preschool population (Hjorth et al., 2016; Groppe and Elsner, 2017; Raine et al., 2018). For instance, Raine et al. (2018) showed that children with obesity significantly underperformed compared to their healthy weight peers in various cognitive functions such as ability, reading, and math skills. Notably, two earlier studies have found significantly slower computer-based reaction times among elementary-aged children with greater body mass index (Gentier et al., 2013; Frazier-Wood et al., 2014). Recent evidence also indicated that over one-third of studied preschoolers were classified as overweight/obese and were more likely to demonstrate motor and cognitive delays (Cataldo et al., 2016; Hamilton et al., 2017). Similarly, another study by Musalek et al. (2017) indicated children (3–6 years old) that were overweight/obese were three times more likely to have severe motor deficits than their non-obese peers. However, observed obesity disparities in motor and cognitive development are still in its infancy and most previous studies implemented paper-and-pencil surveys to subjectively assess cognitive function (Hjorth et al., 2016; Quan et al., 2018), which has been claimed to be lacking process-purity and often paired with outdated principles (Kessels, 2019). More evidence is needed to further investigate the dynamic effects of overweight/obesity may yield on both FMS and cognitive functioning in early childhood to minimize the potential disparities identified in the literature.

Socio-demographic (gender, SES) disparities among FMS have been well documented in the literature; preschool-aged boys have been shown to surpass girls, specifically in ball skills (Goodway et al., 2010; Vameghi et al., 2013; Kokštejn et al., 2017). In one longitudinal study, it was suggested that the magnitude of the gender disparities in FMS (specifically ball skills) could be accelerated from childhood into adolescence (Barnett et al., 2010). Preschoolers living in low SES conditions may also present delayed motor proficiency compared to normative values and/or high SES counterparts (Goodway et al., 2010; Liu et al., 2015). Consequently, these motor delays may transpire into physical inactivity, cognitive dysfunction, and lack of school readiness in later stages of childhood and adolescence (Piek et al., 2008; Stodden et al., 2014; Hamilton et al., 2017). For example, Piek and colleagues found that SES was a significant predictor of motor performance and cognitive function (i.e., intellect) among their sample of 6–11 year old children (Piek et al., 2008). Gender disparities also exist among cognitive function variables, although methodologies have not been consistent which has created inconclusive findings in the early childhood populations. It has been shown in previous studies that preschool and elementary-aged girls produced better cognitive and executive functions than boys (Berlin and Bohlin, 2002; Vuontela, 2003; Mileva-Seitz et al., 2015). Alternatively, in one study, researchers used a manual assessment to measure reaction time (ruler drop test) in children (M_age_ = 5 years old) and found boys had a quicker reaction time than girls (Latorre-Roman et al., 2018). Further investigation of these gender differences in an objective manner among a preschool sample is essential to further develop gender-inclusive intervention strategies for early development and school readiness.

Given the strong prevalence of childhood obesity and its negative impact on children’s overall health and early success, it is critical to use a comprehensive approach to assess developmental status (i.e., motor and cognitive skills) within early childhood and any potential socio-demographic disparities. Obtaining objectively assessed cognitive measures can be challenging in a young population due to the lack of appropriate measures for specific assessments (Blair et al., 2005). It’s also important to note that cognitive function is an umbrella term that houses many different variables that can be used to represent cognitive function. Reaction time is a longstanding method used to examine cognitive function, providing the interpretation of motor and mental response speeds, as well as measures of movement time, reaction time, response accuracy, and impulsivity (Moradi and Esmaeilzadeh, 2017). This can be measured by evaluating individual’s responses with one (simple RT; SRT) or multiple (choice RT; CRT) stimuli (Moradi and Esmaeilzadeh, 2017). The sensitivity of neuropsychological assessments via computerized tests have been explored (Sahakian and Owen, 1992; Fisher et al., 2011; Syväoja et al., 2015). The Cambridge Neuropsychological Test Battery (CANTAB™) has been deemed age-appropriate for young populations (Fisher et al., 2011). Thus, by measuring FMS (Test of Gross Motor Development—3^rd^ edition; TGMD-3) and cognitive function (CANTAB™), the present study aimed to investigate obesity (healthy weight vs. overweight/obese) and socio-demographic (gender, SES) disparities in FMS (locomotor and ball skills) and cognitive function (reaction time [RT] and movement time [MT]), in a sample of preschoolers.

## 2. Materials and methods

### 2.1. Participants

Using a cross-sectional research design, a total of 74 preschoolers (boys = 36; girls = 38; M_age_ = 4.02 years ranging from 3 to 5 years old) were recruited from two childcare centers located in North Texas. There were 49 children (boys = 26; girls = 23; M_age_ = 4.32 years) recruited from Center A, in which the majority of children were identified as African American (42.9%) while others identified as White (33.3%), Hispanic (14.3%), and Asian (9.5%). Children in Center A were enrolled in the Head Start Program and are identified as low-SES. In Center B, there were 25 children (M_age_ = 3.37 years), in which the majority were identified as White (72.7%), while others identified as African American (4.5%), Hispanic (18.2%), and Native American (4.5%). Center B is a private childcare center and children in this center identify with families in middle or upper class. Geographically, both centers shared the same zip code and were in a suburban area. The study protocol was approved by the University’s *Institutional Review Board* and the directors of both childcare centers. Before the data collection, parent consent forms were obtained.

### 2.2. Measurements

#### 2.2.1. Body mass index

Body mass index (BMI) was calculated using children’s height (inches) and weight (lbs) and then categorized into a BMI percentile established from the Centers of Disease Control and Prevention (CDC, 2020). According to the CDC chart, children were then categorized into healthy weight (*n* = 58, BMI percentile 5%–85%) and overweight/obese (*n* = 16, BMI percentile ≥ 85%) groups, respectively.

#### 2.2.2. Fundamental motor skills

Children’s fundamental motor skills (FMS) were assessed using the Test of Gross Motor Development—3^rd^ edition (TGMD-3; Kipling Webster and Ulrich, 2017). The TGMD-3 assesses 13 FMS: six locomotor skills and seven ball skills in children 3–10 years of age. Locomotor skills include running, skipping, galloping, hopping, horizontal jumping, and sliding. Ball skills included two-hand striking, one hand striking, overhand throwing, underhand throwing, kicking, dribbling, and catching. The researcher verbally explained how to perform each skill and demonstrated the skill to each child. Children then performed 2 trials of each skill and were assessed based on each skills’ component criteria (0 = component was unsuccessful; 1 = component was successful). The total score for the locomotor and ball skills subtests are 48 and 54, respectively. Guided by the TGMD-3 gender and age-specified scoring system, children’s raw score for locomotor and ball skills were converted to a standard score (Mean = 10, SD = 3). Psychometric properties have been established in children from the United States (Webster et al., 2015).

#### 2.2.3. Cognitive function

The cognitive domain of attention can measure continuous performance and visual sustained attention. Reaction Time (RTI) from the Cambridge Assessment of Neuropsychological Test Automated Battery (CANTAB™) provides assessments of the psychomotor speed by looking at an individual’s ability to detect and respond to rapid changes in the environment, such as the presence of a stimulus. With this the reaction time and movement time were measured in this study. It is recommended the CANTAB cognitive assessments shall be applied to participants ≥4 years of age, so a subgroup of children aged over 4 were assessed. Eligible children were instructed to hold down the response button with their dominant forefinger on a 10 × 10 inch iPad until the target stimulus was presented (flashed yellow). Once the stimulus was presented, participants would lift their finger, touch the stimulus, then return their forefinger to the response button as quickly and accurately as possible. Reaction time (RT; time taken to release the response button after the presentation of the target stimulus) and movement time (MT; time taken from release of the response button and selection of the target stimulus after flashing yellow on the screen) were calculated for this study. Specifically, single reaction time (SRT) and single movement time (SMT) were calculated in a simple reaction time situation while the child was required to respond to one possible stimulus and then returning to the response button. Choice reaction time (CRT) and choice movement time (CMT) were captured in a choice reaction time situation where the child was required to respond to one of the five possible stimuli and then returning to the response button. The accuracy scores (time in milliseconds) were calculated based on the total correct and assessed trials per child. The average RT and MT (time in milliseconds) in both single and choice reaction time situations were generated through the CANTAB™ software and used in the analyses. Internal consistency and stability have been established in a childhood population using the RTI task (Syväoja et al., 2015).

### 2.3. Data collection and procedures

The researchers performed the data collection during recess and free times in each childcare center. During children’s recess time, research staff went to each childcare center to measure children’s height (inches) and weight (lbs). For assessing FMS, a trained research assistant provided a verbal and kinesthetic demonstration to each child, and then the trained raters (one for locomotor and one for ball skills) graded each child’s performance of each skill after one practice trial. The assessment was conducted outside on the playground or inside the gym (depending on weather). On a separate day (2–4 days apart), the research team assessed children’s cognitive function (i.e., reaction and movement time) via CANTAB™. One member of the research team administered the CANTAB™ test via a 10 × 10 inch iPad individually (~15 min per child) as recommended by the manufacturer.^1^ One trained research member and one child relocated to a private, quiet area where there would be little to no distractions while the CANTAB™ test was administered. A subgroup of children who were 4–5 years old (*n* = 21; 8 boys and 13 girls) performed the CANTAB™ test.

#### 2.3.1. Statistical analyses

Descriptive analyses (minimum, maximum, mean, standard deviation) of BMI percentile, FMS, and cognitive function were conducted. The power analysis (G*Power 3.1.9.4) was conducted for group comparisons in fundamental motor skills. The results indicated the sample size for a group size ratio as low as 0.28 (i.e., overweight/obese group vs. healthy weight group) met the minimum requirements to achieve 80% power for detecting a large effect (effect size *d* = 0.80) at an alpha level of 0.05 in two-tailed independent t-test. Data was only retained for analyses if children completed all components for the RTI and TGMD-3. An independent t-test was used to test the gender, SES, and obesity disparities in FMS and cognitive function variables. Cohen’s *d* was reported for effect size and an alpha level of 0.05 using a two-tail test was incorporated in all analyses. All data analyses were completed using SPSS version 26.

## 3. Results

Descriptive characteristics for BMI percentile, FMS and cognitive function among studied children are shown in Table 1. In total, there were 38 girls and 36 boys between both centers with an average age of 4.02 years (±0.73). There were 58 children classified as healthy weight and 16 classified as overweight/obese. Among the 74 children, 21 were classified as high SES and the remaining were classified as low SES based on their respected preschool. The studied children had a BMI-for-age percentile average of 56.19 (SD = 29.76; ranging from 1.11 to 99.9 percentile). Overall, children showed lower locomotor standard scores (*M* = 7.87 ± 3.77) and ball skill standard scores (*M* = 8.04 ± 2.54) compared to a recent paper examining FMS in 339 preschool aged children (locomotor: *M* = 10.0 ± 0.2; ball skills: *M* = 8.5 ± 0.1) (Kit et al., 2017). Regarding cognitive function (*n* = 21), all preschoolers in this study demonstrated fairly similar responses between single and choice reaction and movement times: SRT (756.81 ± 297.07), SMT (308.12 ± 130.79), CRT (751.45 ± 269.80), and CMT (354.25 ± 172.21). To our knowledge, there were no previous studies that have used this objective measurement among 3- to 5-year-old children, thus, comparisons with other datasets was not applicable.

**Table 1:** Descriptive analysis of weight status, fundamental motor skills, and cognitive function.

Variables	Minimum	Maximum	*M (SD)*
BMI percentile	1.10	99.90	56.19 (29.76)
Ball skills (SS)	2.00	14.00	8.04 (2.54)
Locomotor (SS)	3.00	14.00	7.87 (2.77)
Single RT (ms)	412.72	1432.30	756.81 (297.07)
Single MT (ms)	166.54	767.80	308.12 (130.79)
Choice RT (ms)	425.00	1562.75	751.45 (269.80)
Choice MT (ms)	212.50	1037.40	354.25 (172.21)

BMI, body mass index; SS, Standard Score; SRT, simple reaction time; SMT, simple movement time; CRT, choice reaction time; CMT, choice movement time; M, mean; SD, standard deviation; ms, milliseconds.

**Figure 1:**
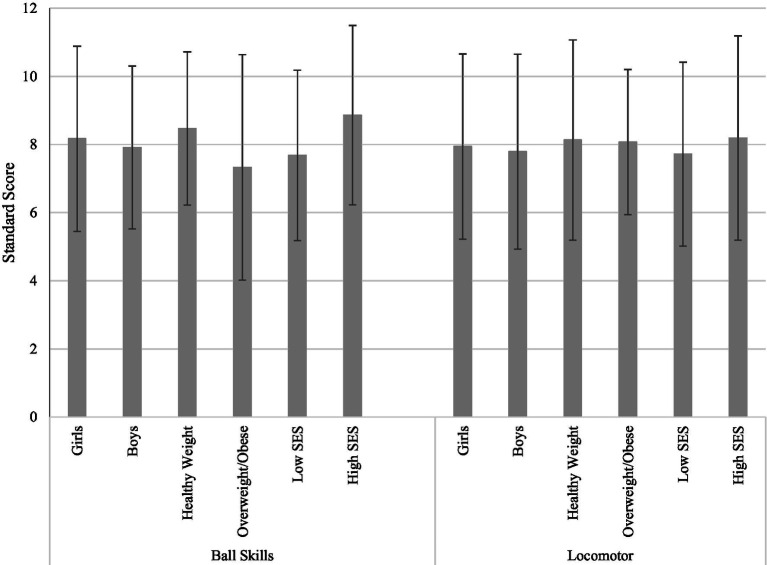
Disparities in fundamental motor skills (*n* = 74). Averages of each demographic group is graphed with standard deviations as error bars. SES, socio-economic status; *p*s > 0.05.

Overall, there were no significant gender differences within BMI percentile (*p* > 0.05), but girls were in a slightly higher percentile compared to boys (*M* = 60.56 vs. *M* = 53.12; Cohen’s *d* = 0.31). Boys and girls also had similar FMS scores in both ball and locomotor categories. The group differences between children in the healthy weight and overweight/obese groups were insignificant in terms of FMS (*p*s > 0.05) even though the healthy weight group marginally outperformed the overweight/obese group in ball skills (8.47 vs. 7.33, respectively). As for SES, there were no significant differences in FMS, however differences in ball skills approached significance, favoring children classified as higher SES, with a moderate effect size (high SES *M* = 8.86 vs. low SES *M* = 7.68; Cohen’s *d* = 0.46). These results are presented in Table 2 and Figure 1.

**Table 2:** Independent *T*-test for fundamental motor skills and cognitive function.

Variables	Gender			Weight status			SES		
	Female	Male	Cohen’s *d*	Healthy weight	Overweight/obese	Cohen’s *d*	Low SES	High SES	Cohen’s *d*
Ball skills (SS)	8.17 (2.72)	7.91 (2.39)	0.10	8.47 (2.25)	7.33 (3.31)	0.40	7.68 (2.50)	8.86 (2.63)	0.46
Locomotor (SS)	7.94 (2.72)	7.79 (2.86)	0.05	8.13 (2.94)	8.07 (2.13)	0.02	7.72 (2.70)	8.19 (3.00)	0.16
Simple RT (ms)	738.96 (240.56)	785.82 (389.08)	−0.15	667.37 (246.77)	1040.90 (309.32)	−1.04	760.65 (346.94)	750.57 (213.84)	0.03
Simple MT (ms)	296.94 (91.49)	326.27 (184.17)	−0.22	283.32 (86.63)	399.49 (211.46)	−1.33	313.31 (146.43)	299.67 (109.47)	0.11
Choice RT (ms)	744.32 (238.83)	763.03 (331.58)	−0.07	675.14 (161.03)	997.22 (417.25)	−0.93	762.60 (320.38)	733.34 (177.24)	0.11
Choice MT (ms)	328.29 (79.87)	396.44 (264.54)	−0.39	312.80 (78.82)	483.32 (314.86)	−1.43	393.48 (206.73)	290.51 (63.04)	0.67

RT, reaction time; MT, movement time; SS, standard score; ms, milliseconds; SES, socio-economic status.

**Figure 2:**
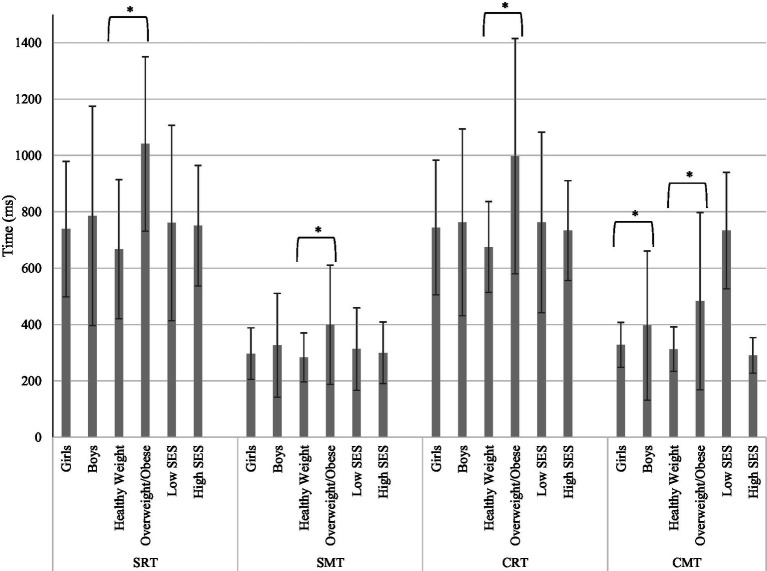
Disparities in cognitive function (*n* = 21). Averages of each demographic group is graphed with standard deviations as error bars. SRT, single reaction time; SMT, single movement time; CRT, choice reaction time; CMT, choice movement time; ms, milliseconds; SES, socio-economic status; *p < 0.05.

Girls (*n* = 13) outperformed boys (*n* = 8) in all four reaction and movement time measures but significantly in CMT (*M* = 328.29 vs. *M* = 396.44; Cohen’s *d* = −0.39). Other cognitive variables (SMT, CRT, and SRT) did not reach significant gender differences (*p*s > 0.05). There were statistically significant differences in SRT, SMT, CRT, and CMT based on children’s weight status (*p*s *<* 0.05). Children categorized into the healthy weight group had faster SMT (*M* = 283.32 vs. *M* = 399.49, Cohen’s *d* = −1.33) and CMT (*M* = 312.80 vs. *M =* 483.32, Cohen’s *d* = −1.43), compared to children in the overweight/obese group. Also, healthy weight children had faster SRT (*M* = 667.37 vs. *M* = 1040.90, Cohen’s *d* = 1.04) and CRT (*M* = 675.14 vs. 997.22, Cohen’s *d* = −0.93) compared to children in the overweight/obese group. It was found that SES showed effects with a moderate effect size on the CMT but not on other tests (high SES *M* = 290.51 vs. low SES *M* = 393.48; Cohen’s *d* = 0.67). These results are visualized in Figure 2 by each measured cognitive variable.

## 4. Discussion

The purpose of this study was to investigate obesity (healthy weight vs. overweight/obese) and socio-demographic (gender, SES) disparities in FMS (locomotor and ball skills) and cognitive function (single and choice reaction time and single and choice movement time) in a sample of preschoolers. Objectively measured cognitive function using the CANTAB™ is an innovative strategy, and to the best of our knowledge, this is one of the first attempts to be implemented in a young preschool sample in conjunction with a process-oriented motor skill assessment. The results of this study provide insight of the potential detrimental effects of unhealthy BMI on cognitive function in preschoolers and highlights the likelihood of socio-demographic disparities in developmental outcomes.

### 4.1. Obesity disparities

The investigation for weight-related effects on cognitive function in early childhood is still in its infancy, however, the current study brings a pioneering measurement to further guide future research. As the most important finding of this study, the obesity disparity in cognitive function was significant in this age group. That is, children with healthy weight had a significantly faster reaction and movement time in a single and choice exhibition compared to their overweight/obese peers. It was noted that obesity can be detrimental to cognitive functioning, however, specific mechanisms are still being uncovered in youth populations (Eichen et al., 2018). Obesity-related biomarkers have been seen to be associated with different areas of cognitive function (i.e., learning, memory, and general ability), but have mainly been observed in animal models and adult populations; identifying the presence of potential biomarkers in an early childhood may be necessary to effectively deter the negative effects on cognitive function in a more proactive and timely manner (Miller et al., 2015). Furthermore, there is limited evidence using neuropsychological assessment of cognitive function, specifically regarding reaction time measurements, so more research with longitudinal designs are suggested to further dissect this relationship in early childhood. Children with healthy weight may have more cognitive advancements and greater academic achievements than children categorized as overweight/obese possibly due to their higher activity/fitness levels (Hillman et al., 2005; Castelli et al., 2007; Davis et al., 2011; Palmer et al., 2013; Crova et al., 2014). Due to obesity potentially positing lower physical activity/exercise levels in children compared to children in a healthy weight category, children that are overweight/obese may not sufficiently activate neurophysiological components in their brain essential for cognitive function (Hillman et al., 2008), which could inevitably hinder their school readiness and success entering elementary/grade school. Two recent reviews examining cognitive functions with physical activity and motor skill performance in a preschool sample underscore the lack of sound methodology in retained studies and encourage future studies to adopt a clinical trial design that emphasizes different components of cognitive function (Jylänki et al., 2022; Malambo et al., 2022). It’s important to mention that the relationship between obesity and physical activity is not linear, so other factors would also need to be accounted for in future studies for a comprehensive understanding. Maintaining healthy weight status in early childhood is essential to promote cognitive function, which ultimately may benefit school readiness when entering elementary/grade school. It is of great benefit for future studies to objectively measure physical activity/fitness levels in conjunction with objective cognitive measures, to understand this relationship more thoroughly in a preschool population.

These results suggest that providing more opportunities for preschoolers to participate in physical activity in and out of the school settings are highly recommended to eliminate the obesity disparity in early childhood. If children are playing organized sports/activities that require timing execution (baseball/softball, dance), it’s possible these activities are not only promoting a healthier BMI, but also improving their cognitive skills (i.e., reaction and movement timing). A recent study found that using an intervention containing motor games (motor stories, learning environments, and motor circuits) can be used to facilitate learning new skills, such as swimming (Simón-Piqueras et al., 2022). Although assessing physical activity and/or fitness was outside the scope of this study, research has shown in school-aged children, more physical activity/greater fitness was related to greater cognitive gains (Castelli et al., 2007; Hillman et al., 2008). The overweight/obese group in our sample may not have participated in organized sports/activities compared to their healthy weight peers, which could deter important physiological and metabolic responses conducive for healthy cognitive function (Hillman et al., 2008). Assessing cognition objectively (i.e., reaction and movement time via CANTAB™; investigating brain activity) in a young preschool sample may be needed in future work to identify delays or signs of dysfunction earlier so appropriate interventions could be implemented sooner (i.e., structured physical activity or FMS-based recess). Previous research also indicates that different types of physical activities for obesity prevention could influence (hinder or improve) children’s cognitive functioning from a young age. For example, activities such as playing outside, playing strategy-based sports/games, and active classroom breaks may instantly activate the brain and promote reaction time capability, whereas passive sedentary activities (i.e., only watching TV/video, using internet on mobile or computer device) may hinder or delay cognitive development (Esteban-Cornejo et al., 2015). Since these children are only 3–5 years old, it’s unlikely that they have developed autonomy over their free time, so their activities may be determined from their parents/family’s interests and values. Early childhood obesity prevention strategies may also be required to be individually tailored towards community and home environmental specific structures to fully capture a child’s developmental profile (Moss and Gu, 2022).

The current findings are consistent with two previous studies among preschoolers; however, methods of cognitive assessments differed (Krombholz, 2013; Martin et al., 2016). For instance, Krombholz (2013) assessed intelligence, verbal ability, and concentration to represent cognition and found children in the overweight group demonstrated lower intelligence. Martin et al. (2016) assessed vocabulary, picture similarity, and pattern construction to represent cognition and reported that obesity at 3 years old was associated with some significant adverse cognitive outcomes at 5 years old, robustly in boys’ pattern construction. Even though a similar trend favoring healthy weight was reached among these studies, assessing cognition utilizing various methodology may make generalizing results difficult, especially in a young sample. There is a need for more standardized, objective measurements in younger samples’ cognitive function to produce more comparable data. Nonetheless, the current study augments the established evidence that weight status (favoring healthy weight) influences children’s cognitive function in early childhood. The obesity disparity revealed here should encourage schools and parents to monitor children’s weight status to pave opportunities for cognitive growth.

Although, no significant overweight/obese disparities were observed in FMS, children in the healthy weight group demonstrated better locomotor and ball skills than their counterparts, especially in ball skills. Consistent with a recent systematic review, the relationship between weight status and FMS is inconsistent across age groups and many studies utilized various methods of obesity measures (BMI, Waist circumferences, DXA) (Slotte et al., 2017). Emerging research acknowledges the important role FMS posits toward obesity trajectory during childhood (Stodden et al., 2008; Robinson et al., 2015). The current study provides the preliminary evidence on an emerging obesity disparity trend in ball skills regardless of gender and SES status. It is critical to implement intervention programs that focus on promoting movement opportunities (regularly scheduled FMS-based play time or structured recess) in early childcare settings to ensure sufficient practice and developmental opportunities are provided for this vulnerable population.

### 4.2. Gender disparities

Regarding cognitive function, gender differences were not found through our measures. Girls did, however, show to be faster in reaction and movement time (significantly in CMT) which contradicts the work from Latorre-Roman et al. (2018), exhibiting 4 year old girls demonstrating slower reaction times than boys in the ruler drop test. Within this young population, it’s important to emphasize activities to facilitate healthy cognitive development for both boys and girls. Typically, boys are more involved in organized sports than girls (Marques et al., 2016) which could enhance aspects of cognitive function (i.e., reaction and movement time) due to practicing the skills necessary to complete the action needed in the sport (i.e., timing in throwing, striking). In the current sample, however, girls showed to be faster than boys in both reaction and movement time; specifically, girls presented higher scores on the ball skills involving eye-hand coordination capability. These girls could be involved in extracurricular activities promoting these skills, which could be the potential explanation of the inconsistency with Marques et al.’s findings.

Gender-inclusive FMS interventions have been shown to improve ball skills in girls, which can aid in eliminating the gender disparity at a young age (Veldman et al., 2017). Incorporating more gender-inclusive FMS-based interventions in childcare and home settings can help develop FMS, and consequently, facilitate academic and cognitive success (Chang and Gu, 2018). As a whole, the sample in the current study acquired lower locomotor and ball skills compared to a nationally representative sample (Kit et al., 2017). Childcare center professionals and parents should facilitate gender inclusive opportunities throughout early childhood to further develop FMS and avoid potential motor delays that may enlarge the disparity in later childhood and adolescence.

### 4.3. Socio-economic status disparities

Encompassing the environmental role that is used to shape children’s cognitive function and motor acquisition is essential to fully understand the impact of affordances throughout development (Zoghi et al., 2019). Although SES was not seen to bear significant differences in cognitive function, it’s important to note there were observed slower reaction and movement times throughout the low SES group as well as the overweight/obese group. Families that are identified as low income may struggle to support the fundamental needs of their children and may not have available funds to invest in cognitive stimulating activities/opportunities as wealthier families would (Poh et al., 2019). It has been shown cognitive function is linearly associated with parental education (Noble et al., 2015), which is a correlate of SES. Low SES preschoolers in the current study may not have the same affordances for cognitive growth at home or school settings as their high SES peers may have, which could explain the emerging disparity in cognitive function.

It was found that children identified as low SES demonstrated poorer locomotor and ball skills compared to children identified as high SES, even though disparities did not reach significance. The SES and gender disparities in preschoolers’ FMS and cognitive development may even widen or become statistically significant due to the COVID-19 pandemic by exacerbating the already known health-related disparities in low SES populations (López-Bueno et al., 2020). Pairing that with childcare/school closures throughout 2020 and 2021 might lead detrimental effects on low SES children’s motor development due to the lack of motor affordances they may have in their home environment. Future research should investigate the implications of the COVID-19 pandemic on preschoolers’ developmental outcomes, especially in vulnerable populations such as children in low SES and/or overweight/obese children. It’s important for childcare professionals to provide opportunities fairly and adequately for motor and cognitive growth, such as incorporating different toys and equipment in their curriculum and provide more opportunities to promote equality of affordances, especially in the low-SES population. Public policy and government funding strategies may need to be reevaluated to ensure these needs can be met.

## 5. Conclusion

The strength of this study was assessing cognitive function objectively using CANTAB™ in a preschool-aged sample and identifying potential obesity and socio-demographic disparities in motor and cognitive outcomes. The CANTAB™ assessment of reaction and movement time provides solid, preliminary evidence of the status of children’s cognitive function and also generates insights of how socio-demographic factors may influence FMS and cognitive function. Some limitations should be acknowledged, however. First, all children lived a similar geographic region, which can make it difficult for generalizability of the findings. Second, given the strength of the objective measures for cognitive function, we were only able to assess a small subgroup of children in this study due to its monetary and timely costs as well as the guideline from CANTAB™. The current findings highlight the importance of healthy weight status in young children’s development, especially regarding cognitive function. It’s important for parents and childcare stakeholders to actively monitor weight status and encourage cognitively-stimulating activities that will promote motor skill development and also cognitive functions such as reaction and movement time. In conclusion, this study provides preliminary evidence identifying the potential obesity and socio-demographic disparities in preschoolers’ motor and cognitive skill development using objective measurements during early childhood.

## Data availability statement

The raw data supporting the conclusions of this article will be made available by the authors, without undue reservation.

## Ethics statement

The studies involving human participants were reviewed and approved by University of Texas at Arlington IRB. Written informed consent to participate in this study was provided by the participants’ legal guardian/next of kin.

## Author contributions

All authors listed have made a substantial, direct, and intellectual contribution to the work and approved it for publication.

## Funding

The authors received financial support for this research from the University of Texas at Arlington Research Enhancement Program (REP).

## Conflict of interest

The authors declare that the research was conducted in the absence of any commercial or financial relationships that could be construed as a potential conflict of interest.

## Publisher’s note

All claims expressed in this article are solely those of the authors and do not necessarily represent those of their affiliated organizations, or those of the publisher, the editors and the reviewers. Any product that may be evaluated in this article, or claim that may be made by its manufacturer, is not guaranteed or endorsed by the publisher.

## Footnotes

^1^ http://wwww.cantab.com
